# Folate Functionalized Lipid Nanoparticles for Targeted Therapy of Methicillin-Resistant *Staphylococcus aureus*

**DOI:** 10.3390/pharmaceutics13111791

**Published:** 2021-10-26

**Authors:** Kushal Vanamala, Ketki Bhise, Hiram Sanchez, Razieh Kebriaei, Duy Luong, Samaresh Sau, Hosam Abdelhady, Michael J. Rybak, David Andes, Arun K. Iyer

**Affiliations:** 1 Use-Inspired Biomaterials & Integrated Nano Delivery (U-BiND) Systems Laboratory, Department of Pharmaceutical Sciences, Eugene Applebaum College of Pharmacy and Health Sciences, Wayne State University, Detroit, MI 48201, USA; gq1789@wayne.edu (K.V.); ketki.bhise@wayne.edu (K.B.); duy.luong@wayne.edu (D.L.); gi7517@wayne.edu (S.S.); hosamabdelhady@wayne.edu (H.A.); 2 Division of Infectious Disease, Department of Medicine, University of Wisconsin-Madison, Madison, WI 53706, USA; hxs@medicine.wisc.edu (H.S.); dra@medicine.wisc.edu (D.A.); 3 Anti-Infective Research Laboratory, Eugene Applebaum College of Pharmacy and Health Sciences, Wayne State University, Detroit, MI 48201, USA; r.kebriae@wayne.edu (R.K.); m.rybak@wayne.edu (M.J.R.); 4 Lake Erie College of Osteopathic Medicine, Erie, PA 16509, USA; 5 Molecular Imaging Program, Karmanos Cancer Institute, Detroit, MI 48201, USA

**Keywords:** vancomycin, liposomes, MRSA, thigh infection, antibacterial resistance

## Abstract

Methicillin-resistant *Staphylococcus aureus* (MRSA), commonly called a superbug, is a highly alarming antibiotic-resistant population of *Staphylococcus aureus (S. aureus)* bacteria. Vancomycin (VAN) was first approved by the FDA in 1988, and it is still regarded as the treatment of choice for MRSA. The efficacy of VAN treatment has become less effective due to the development of VAN resistance in MRSA and the potential for nephrotoxicity. This study aims to improve the efficacy of VAN treatment by identifying the folate receptor for MRSA infected tissues and developing folate decorated lipid nanoparticles containing VAN (LVAN). In comparison to conventional VAN, LVAN showed a higher bactericidal effect and a superior ability to inhibit biofilm in MRSA with an enhanced accumulation in MRSA infected thigh tissues and a reduced accumulation in kidney. The results suggested that LVAN is a promising candidate to overcome the current limitations of bacterial resistance and adverse side effects in kidneys found in VAN.

## 1. Introduction

*Staphylococcus aureus* is a common human pathogen that normally dwells on the skin. Methicillin-resistant *Staphylococcus aureus* (MRSA) is a strain responsible for infections with limited, often ineffective treatment options due to the emergence of antibiotic resistance [1]. While MRSA most commonly causes skin infections in the community setting, in healthcare settings it may cause infections of the bloodstream, surgical site infections, or pneumonia. In serious cases, it may lead to sepsis and death. In the US alone, annually, around 120,000 patients are diagnosed with, and 20,000 succumb to MRSA [2,3]. Globally MRSA infected patient numbers increased to approximately 400,000 [4]. Infections caused by MRSA in both community and hospital settings is thus a matter of concern [5].

Vancomycin (VAN) is regarded as the treatment of choice for MRSA [6]. However, there are limitations with VAN treatment for MRSA in terms of low tissue penetration, slower bactericidal activity, drug resistance, and the potential for toxicity [7,8]. Higher doses of VAN is associated with nephrotoxicity (defined as 0.5 mg/dL serum creatinine levels, or 50% higher than the baseline) in a sizeable subset of adult patients [9]. Strategies to overcome the toxic side-effects of VAN, while maximizing deliverable dose and in turn, improving drug efficiency, may be helpful to establish a newer clinical therapy regimen of VAN. In terms of antimicrobial drug delivery, it is essential to deliver the drug to the infected tissue at a higher concentration, while penetrating through a rigid biological biofilm barrier that imparts drug resistance and microbial survival. Biofilm-producing *Staphylococcus aureus* tends to form a cluster of colonies on medical devices and catheters and may trigger serious infections [10]. Treatment failure in drug-eluting medical devices or prosthetics can be attributed to a large extent to the development of microbial biofilms that lower antimicrobial susceptibility and pose a challenge to drug penetration through the biofilm matrix [11]. In addition, it is well established that antibiotic therapy comes at a cost of negatively altering the human microbiota, resulting in adverse health issues [12].

In this regard, it has been demonstrated that targeted nanoparticles loaded with antibiotics protected the composition of fecal microbiota in a mouse model, while showing a desired therapeutic response for *S. aureus* infection [13]. Liposomes have gained widespread attention due to their clinical success for several diseases including cancer, pain management, fungal and viral infections [14,15,16]. Liposomal formulation of amphotericin-b (AmBisome^®^) showed a significantly reduced nephrotoxicity than conventional amphotericin-b [14]. Clinically-translatable lipid nanoparticle delivery systems with particle sizes ranging from 80 to 300 nm offer several advantages over conventional free drug administration, such as (1) protecting the antibiotic payload from degradation or release during transit; (2) limiting the antibiotic exposure to non-target organs and tissues, leading to higher safety; (3) enhancing antibiotic half-life and plasma circulation, improving bioavailability and retention; and (4) improving the selective accumulation at sites of infection [15,17,18,19,20]. 

Earlier we had reported a novel method for encapsulating highly water-soluble drugs (VAN and cefazolin) in passively targeted liposomes with a markedly high drug loading (US patent # 62/612,191) [21]. In this study, we have demonstrated the antimicrobial efficacy of folate-targeted VAN liposomes synthesized using our reported method, in MRSA biofilm and animal models. Literature evidence suggests that folate receptor (FR) has a leading role in the induction of pro-inflammation, in MRSA infected sites [21,22,23]. In this study, we, for the first time, determined the expression of FR in *S. aureus*-infected animal tissues. We found that FR expression is significantly increased in MRSA-infected animal tissue compared to uninfected control tissues. We successfully developed folate-targeted VAN liposomes and characterized them for physicochemical properties such as particle size, zeta potential, atomic force microscopy, release kinetics. Following this, the liposomes were tested for antimicrobial efficacy and inhibition of biofilm in vitro. The in vivo testing in the MRSA-infected animal model demonstrated superior uptake of the liposomes at the site of infection while minimizing localization in the liver and the kidneys. Lastly, the VAN-loaded folate targeted liposomes were able to arrest microbial growth and promote a reduction in microbial load in an MRSA mouse thigh infection model. The results suggested that the novel folate-targeted VAN liposomes could be a new strategy to solve the limitation of conventional treatment of VAN with a lower MIC, a higher tissue penetration, and a lower accumulation in the kidney.

## 2. Materials and Methods

### 2.1. Materials

Hydrogenated soybean phosphatidylcholine (HSPC) was purchased from NOF Corporation (Tokyo, Japan). Moreover, 1,2-distearoyl-sn-glycero-3-phosphoethanolamine with conjugated methoxyl poly(ethylene glycol) (DSPE-mPEG_2000_), 1,2-distearoyl-sn-glycero-3-phosphoethanolamine with conjugated poly(ethylene glycol)-folic acid (DSPE-PEG_2000_-FA) was purchased from Nanocs Inc. (Boston, MA, USA). Cholesterol was purchased from Sigma-Aldrich (St. Louis, MO, USA). VAN was purchased from Alfa Aesar (Haverhill, MA, USA). Sterile phosphate-buffered saline (PBS; Thermo Fisher Scientific, Waltham, MA, USA) pH 7.4 was used for preparing liposomes. All organic solvents were purchased from Thermo Fisher Scientific (Waltham, MA, USA) and were of 99.5% purity. Bacterial strains MRSA 494 and MSSA ATCC 29213 were obtained from the Anti-Infective Research Laboratory (Detroit, MI, USA). Mueller-Hinton broth (Difco, Detroit, MI, USA) supplemented with 25-mg/L calcium, 12.5-mg/L magnesium was used for all microdilution susceptibility testing and time-kill analyses. Trypticase soy agar (Difco, Detroit, MI, USA) was used for growth and quantification of organisms.

### 2.2. Immunohistochemistry for Folate Receptor Expression

MRSA-infected eye tissues harvested from mice were used to study the expression of folate receptors. Immunohistochemistry (IHC) was performed on FFPE tissue slides, which were a kind gift from the lab of Dr. Ashok Kumar (Kresge Eye Institute, Detroit, MI, USA). The tissues were incubated with anti-folate binding protein (Abcam, MA, USA) at a dilution of 1:1000 with PBST overnight at 4 °C. The tissues were then washed with PBST and counterstained with anti-rabbit FITC (Invitrogen, Waltham, MA, USA) at a dilution of 1:1000 with PBST and incubated at room temperature under mild shaking for 2 h. Folate receptor expression was studied using fluorescence microscopy at a magnification of 20×.

### 2.3. Formulation Development of LVAN

VAN liposomes (LVAN) were prepared using the method reported earlier by our group for achieving high drug loading of water-soluble drugs in liposomes [21]. Briefly, lipid mixture of HSPC: cholesterol: DSPE-mPEG_2000_: DSPE-PEG_2000_-FA in the ratio of 1: 0.05: 0.007: 0.0001 was dissolved in an equal proportion of diethyl ether: chloroform. The solvents were gradually evaporated under vacuum to form a thin film of lipid, which was redissolved in the same solvents. To this, an equal volume of sterile PBS-containing VAN was added dropwise with rigorous mixing, followed by gradual evaporation of the volatile solvents under a high vacuum. Unencapsulated VAN was removed by filtration (Amicon^®^ Ultra Centrifugal Filters, Millipore Sigma, Burlington, MA, USA; MWCO 3.5 kDa), followed by 10 cycles of high-pressure homogenization at 150,000 kPa. VAN liposomes (LVAN) thus formed were analyzed for drug loading and encapsulation efficacy by RP-HPLC using the following formula:

$$\%\;Drug\;loading=\frac{\left(Weight\;of\;drug\;contained\;in\;the\;system\right)}{\left(Total\;weight\;of\;the\;drug\;loaded\;liposomal\;vesicle\right)}\times 100$$

$$\%\;Encapsulation\;efficacy=\frac{\left(weight\;of\;drug\;contained\;in\;the\;system\right)}{\left(Total\;weight\;of\;drug\;loaded\;in\;the\;system\right)}\times 100$$

### 2.4. Physicochemical Characterization

#### 2.4.1. Size, Polydispersity Index, and Zeta Potential

Average particle size, polydispersity index, and zeta potential of the liposomes were analyzed using dynamic light scattering (DLS, Beckman Coulter DelsaNano CTM equipped with a 658 nm He-Ne laser, Beckman Coulter, Indianapolis, IN, USA). The samples were diluted 20-fold to 1.5 mL with deionized water and the scattered light was detected at an angle of 165°. The software used for sample analysis was provided by the manufacturer (Beckman Coulter, DelsaNano version 2.2, Indianapolis, IN, USA). The size and morphology of LVAN were studied using Transmission Electron Microscopy (TEM, H-7500, and Hitachi Ltd., Tokyo, Japan). Samples were obtained by placing appropriately diluted nanoparticles onto a carbon-coated 200 mesh copper grid to form a thin film. The film was stained with uranyl acetate, and the excess staining solution was removed with filter paper.

#### 2.4.2. Atomic Force Microscopy

The AFM images were done on a Veeco Dimension Icon Atomic Force Microscope (Bruker, Santa Barbra, CA, USA). Silicon nitride sharpened probes with nominal tip radius R = 2–12 nm (Bruker, Santa Barbra, CA, USA) were used. All images were conducted in the air by using the Peak Force Quantitative Nanomechanical (PFQNM) mode. Scan rates of 1–2 Hz were applied. Freshly cleaved, atomically flat mica (Agar Scientific, Essex, UK) was first scanned in DI water to confirm the absence of contamination and then was used as a substrate. Post-imaging analysis was done on a NanoScope 8.1 software (Bruker, Santa Barbra, CA, USA). Images were flattened to remove sample tilt; probe shape deconvolution was made using SPIP software (version 6.7.1, Image Metrology Lyngby, Denmark).

#### 2.4.3. In Vitro Drug Release Study

In vitro drug release study was performed using dialysis method modified in-house, as reported by us in [21]. Briefly, 500 μL of LVAN was introduced to Slide-A-Lyzer™ Mini dialysis device, 3.5 kDa MWCO (Thermo Fisher Scientific, Waltham, MA, USA), which was plugged into a 1.5 mL microcentrifuge tube (Thermo Fisher Scientific, Waltham, MA, USA) containing 1.5 mL of PBS pH 7.4 as release buffer. The assembly was placed in a water bath on a magnetic stirrer (Thermo Fisher Scientific, Waltham, MA, USA) at 350 rpm, 37 °C. Aliquots of 100 μL were removed from the release buffer over 72 h. An equal volume of release buffer was replaced at every time point to maintain sink conditions. The aliquots were analyzed by HPLC to quantify VAN released in the buffer. The graph of % cumulative release versus time (hours) was plotted (*n* = 2).

### 2.5. Susceptibility Testing

Minimum inhibitory concentrations (MIC) values for VAN and LVAN were determined in duplicate by standardized broth microdilution techniques with a starting inoculum of 5 × 10^5^ CFU/mL, according to the Clinical and Laboratory Standards Institute guidelines reference. All samples were incubated for 18 to 24 h at 35 °C.

### 2.6. In Vitro Disk Diffusion Assay

Disk diffusion assay for VAN vs. LVAN was determined in duplicate by the Kirby–Bauer procedure, with an inoculum of 150 million CFU/mL, in 4 mm deep Mueller-Hinton agar poured into Petri dish. The pH of agar was maintained at 7.2–7.4 and all samples were incubated for 24 h at 35 °C.

### 2.7. Biofilm Inhibition of LVAN vs. VAN

CDC biofilm reactor (CBR) model (BioSurface Technologies, Bozeman, MT, USA) was set up with 24 polyurethane coupons inserted into eight rods (3 coupons per rod). A 40 h biofilm conditioning phase was performed prior to evaluation of the antimicrobials and consisted of a 24 h incubation at 37 °C of inoculated 1% gSTSB, followed by 16 h of continuous flow with 10% gSTSB performed with peristaltic pumps (Masterflex; Cole-Parmer Instrument Co., Chicago, IL, USA), set up at a rate of 13.3 mL/min to achieve a 30-min residence time. Once the continuous flow phase was completed, Mueller-Hinton broth was utilized as the medium for the model experiments. Boluses of VAN and LVAN were injected into the reactor after the biofilm conditioning phase was completed. Peristaltic pumps were then set up to simulate the half-lives of the antibiotics. VAN and LVAN regimens were evaluated at q12h. Each model was evaluated in duplicate to ensure reproducibility.

### 2.8. In Vivo Testing on Infected Mouse Thigh Model

The neutropenic murine thigh infection model was used for in vivo study of VAN [24,25,26]. Animals were maintained in accordance with the American Association for Accreditation of Laboratory Animal Care (AAALAC). All animal studies were performed under Dr. David Andes animal protocol#DA0055, approved on 14 July 2021 by the Animal Research Committees of the William S. Middleton Memorial VA Hospital and the University of Wisconsin, Madison. 

Murine thigh infection model experimental detail: *Animal species*: 24–27 g CD-1 (Swiss ICR) mice from Envigo were used for all studies. In this regard, we utilized both genders. *Induction of neutropenia*: mice were rendered neutropenic (polymorphonuclear cell count, <100 mm^3^) by two intraperitoneal (I.P) injections of cyclophosphamide at 150 mg/kg of body weight at 4 days and 100 mg/kg at 1 day before infection. *Organism preparation*: the bacterial isolates were sub-cultured from frozen stock on MH agar. Colonies were collected and grown in MH broth using a shaking incubator at 37 °C until log-phase growth. The bacterial cells were collected by centrifugation, washed, and diluted in phosphate-buffered saline (PBS) to achieve the desired starting inoculum as determined by optical density (OD) measurement. The inoculum were confirmed by plating serial dilutions onto MH agar plates and quantifying the number of viable colony-forming units. The typical inoculum for the thigh model is 10^6−7^ CFU/mL. *Infection*: thigh infections were produced by injection of 0.1 mL of each inoculum (10^6^ CFU/mL) into the thighs of isoflurane-anesthetized mice 2 h before antimicrobial therapy. This study design results in log phase growth in the thighs two hours after infection with an organism burden near 10^6^ CFU/thigh. In control animals, the disease model is progressive based on an increase in pathogen load (2–3 log10) by 24 h. *Drug administration*: LVAN-A and VAN were administered by intraperitoneal dosing. The dose range and frequency of administration for LVAN-A and VAN were 0.3125 and 80 mg/kg/6 h. *Treatment regimens*: each arm consisted of the following groups of mice: (1) control animals that received sterile normal saline, using the same volume, route, and schedule as those for the active-treatment regimens. Untreated controls; (2) LVAN and commercial VAN; and (3) positive comparator. Endpoints: at the start of therapy (2 h after infection) and the end of therapy (24 h), all animals were euthanized by carbon dioxide asphyxiation. After sacrifice, the thighs of mice were removed and individually homogenized in normal saline. Ten-fold serial dilutions of the thigh homogenates were plated and incubated overnight at 37 °C. Counts for each thigh were considered independent observations. This microbiologic endpoint is predictive of animal survival but reduces animal number needed for statistical relevance by more than 50%. Efficacy was calculated as the change in the log10 CFU obtained for antibiotic-treated mice at 24 h compared to the pre-antibiotic baseline CFU measured for the control animals at the start of therapy. Animal numbers: two mice were used for each dose level or dosing regimen. This provides four independent thigh infections, which has been typically sufficient to account for variability in the infection model.

### 2.9. In Vivo Tissue Uptake of LVAN vs. VAN

A total of 1 mmol of liposomes encapsulated with rhodamine-conjugated VAN (rhodamine-LVAN) were prepared with the aforementioned method. Mice were injected with a single dose of 50 nmol of rhodamine-LVAN through i.p. administration after 2 h of bacterial inoculation. Mice were euthanized 24 h post-injection; organs were harvested and processed for sectioning on paraffin-embedded glass plates. The processed tissues were imaged under fluorescence microscopy to evaluate tissue uptake of rhodamine-LVAN and rhodamine-VAN in vital organs.

### 2.10. In Vivo Antibacterial Efficacy Study

VAN and LVAN injections were prepared in PBS, with doses from 0.3125 to 80 mg/kg. The mice were injected with the drugs 2 h post-bacterial inoculation intraperitoneally, after every 6 h. The bacterial burden was determined at the start and end of therapy over a 24 h experiment duration. The dose-response curves were plotted for both LVAN and VAN for MSSA (SA 29213) and MRSA (SA 33591) thigh infection models.

## 3. Results and Discussion

### 3.1. Folate Receptor Expression

**Figure 1:**
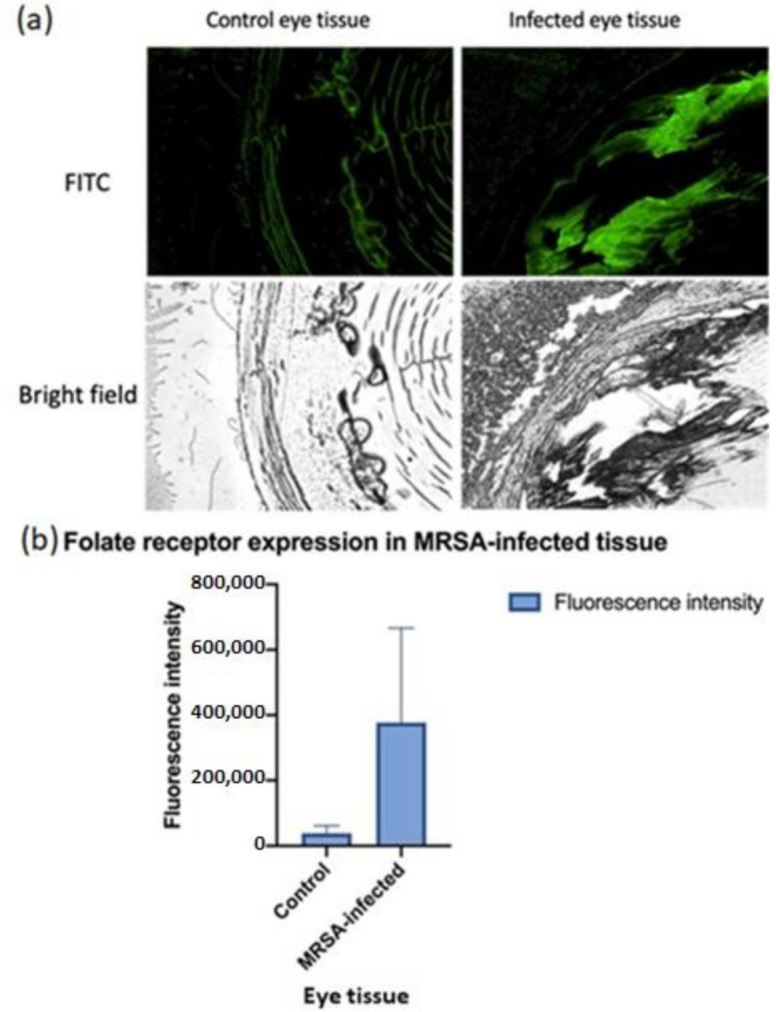
(**a**) Folate expression in MRSA infected tissue. A fluorescent microscopy study showed an overexpression of folate receptor (FR) in MRSA infected tissue in comparison to healthy tissue control. (**b**) The quantification of the folate expression data is shown (*n* = 3). The significantly higher fluorescence intensity of FR in MRSA-infected tissues as compared to healthy tissues builds a strong rationale for developing FR-receptor homing nano-antibiotics for targeted MRSA delivery.

Currently, there are limited biomarkers for infection site-specific delivery. Identification of novel biomarkers for infection site-specific delivery is an important goal that could lead to improvement of antibiotic delivery efficiency, efficacy, and reduction of off-target effects or toxicities. Literature evidence suggests that FR has a leading role in the induction of pro-inflammation, in MRSA infected sites, suggesting that FR could be a biomarker for infected sites [22,23,27]. IHC studies with MRSA-infected tissues showed a marked increase of fluorescence intensity associated with folate receptor expression in MRSA-infected tissues compared to control of healthy tissues. This study solidifies our rationale for the formulation of folate-receptor targeted liposomes that can be selectively transported to the site of MRSA-infected tissues in vivo and endocytosed via receptor-mediated uptake as well as reduce the off-target accumulation in the kidney, which is believed to be associated with acute kidney injury found in VAN treatment [28,29,30]. Figure 1 shows the FITC staining intensities for control versus infected tissues after blocking with the antibody for folate binding protein.

### 3.2. Formulation Development and Physicochemical Characterization

#### 3.2.1. Size and Morphology

**Figure 2:**
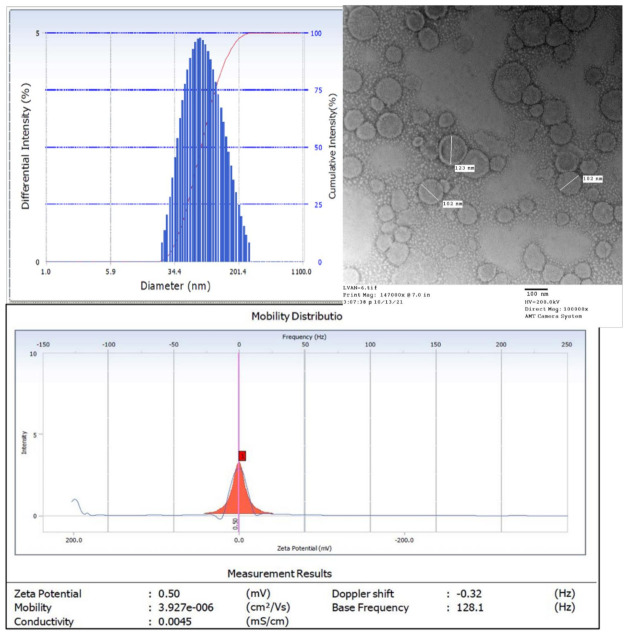
Uniform and homogenous nanosize distribution of LVAN as shown by DLS (top left), bilayer morphology of liposomal vesicles as shown by TEM (top right, scale bar 100 nm), and zeta potential of LVAN (bottom). Data indicate homogenous and nanosized particles, with near neutral surface charge, that are suitable for MRSA targeting.

It has been reported that nanoparticles offer improved penetration through the dense bacterial colonies due to longer retention in plasma and higher interaction of nanoparticles with the lipophilic bacterial environment [31,32]. Our lab has developed a robust method for encapsulation of highly water-soluble drugs in nanosized liposomes, which was optimized to ensure reproducible batches. Accordingly, we achieved a drug loading of 35.3% (*w*/*w*) which was used for in vitro and in vivo testing on infection models. We used a relatively inexpensive technique for particle size reduction, high-pressure homogenization, in place of the classical membrane-extrusion technique, that yielded homogenous batches of liposomes with uniform particle size. Homogenization yielded smaller, unilamellar liposomes due to attrition of multilamellar liposomes subjecting to high pressure. Unilamellar, phospholipid bilayer liposomal vesicles with homogeneous size distribution (reflected by PDI = 0.2) and average diameter between 130 and 160 nm (reflected by TEM images) were prepared using this method. The zeta potential of LVAN was 0.5 mV, which may affect the agglomeration and stability of LVAN in the solution. Zeta potential of roughly 30 mV and above is reported to contribute to physical stability, whereas a zeta potential of less than 20 mV shows limited colloidal stability (Figure 2) [33].

#### 3.2.2. Atomic Force Microscopy

**Figure 3:**
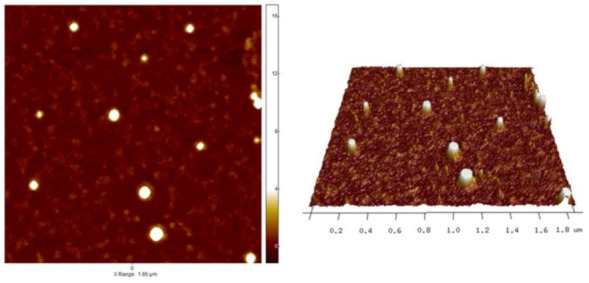
AFM image of LVAN in PBS pH 7.4. The image shows nano-sized particle and uniform distribution of the liposomal formulation.

Figure 3 shows an AFM image of LVAN in 1 mM PBS where spherical features of LVAN were seen. The average measured diameter and height of these features were 66.23 ± 20.82 nm and 6.14 ± 4.28 nm, respectively. The dissimilarity of the measured diameters relative to the measured heights could be attributed to the flattening of the loaded liposomes on the substrate. This flattening will increase the contact area between the liposomal membrane and the substrate. Hence, facilitating the liposomal contact with MRSA membrane and hence increasing the killing efficiency of the liposomal nanoparticles. The image size = 1.85 µm and the Z range = 18 nm.

#### 3.2.3. In Vitro Drug Release

**Figure 4:**
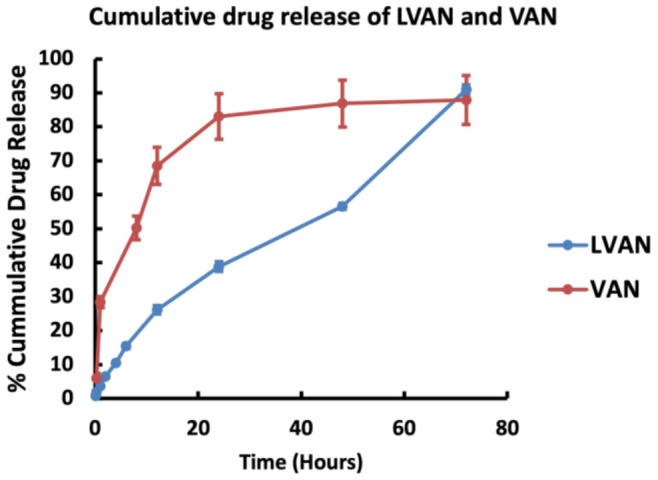
Comparative cumulative drug release profile of LVAN over 72 h shows a controlled VAN release pattern.

To mimic intravenous conditions for injectable dosage of VAN, in vitro drug release study was performed at a pH of 7.4 and the release profile of LVAN was evaluated in comparison to pure VAN as the control. LVAN showed a controlled release pattern over 72 h, with a maximum of 91% drug release compared to VAN, which showed around 82% within 24 h (Figure 4).

### 3.3. Susceptibility Testing

The MIC values for LVAN versus commercial VAN are listed in Table 1. As can be seen from the results, the liposomal VAN MIC values were comparable to that of the commercial product, indicating the need to validate the superior antibacterial activity in the animal model.

### 3.4. In Vitro Disk Diffusion Assay

As shown in Table 2, there was a slight increase in the zone of inhibition for LVAN compared to VAN in strains 494 and 29213. There was no significant difference in the zone of inhibition for LVAN and VAN in strain N315. Larger zone of inhibition corresponded to superior antimicrobial activity for LVAN compared to VAN in vitro.

### 3.5. Biofilm Inhibition of LVAN vs. VAN

**Figure 5:**
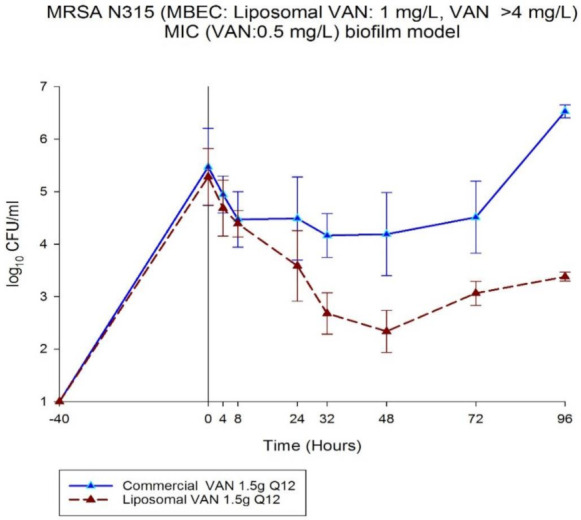
Superior in vitro bactericidal effect of LVAN in comparison to free VAN in MRSA N315 biofilm model is shown.

Figure 5 shows the enhancement in bacterial colony reduction on LVAN treatment as compared to VAN, in MRSA N315 in vitro biofilm model. Overall, LVAN was able to demonstrate better killing activity compared to VAN over a period of 96 h against MRSA strain N315. LVAN was able to show bactericidal effect (−3 log10 CFU/mL from baseline q12h) at 48 h. More pronounced effects might be observed as VAN is demonstrating an upward trajectory of growth after 72 h. A possible reason for the higher efficacy of LVAN compared to VAN in biofilm inhibition could be the lipophilic nature of liposomes that may have aided in penetration of the molecules across the biofilm layer that is rich in polysaccharides and glycopeptides.

### 3.6. In Vivo Testing on Infected Mouse Thigh Model

#### 3.6.1. In Vivo Tissue Uptake of LVAN vs. VAN

**Figure 6:**
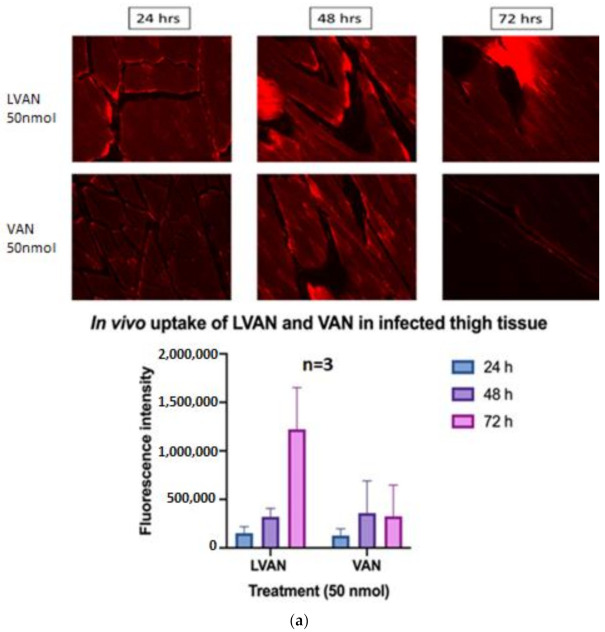

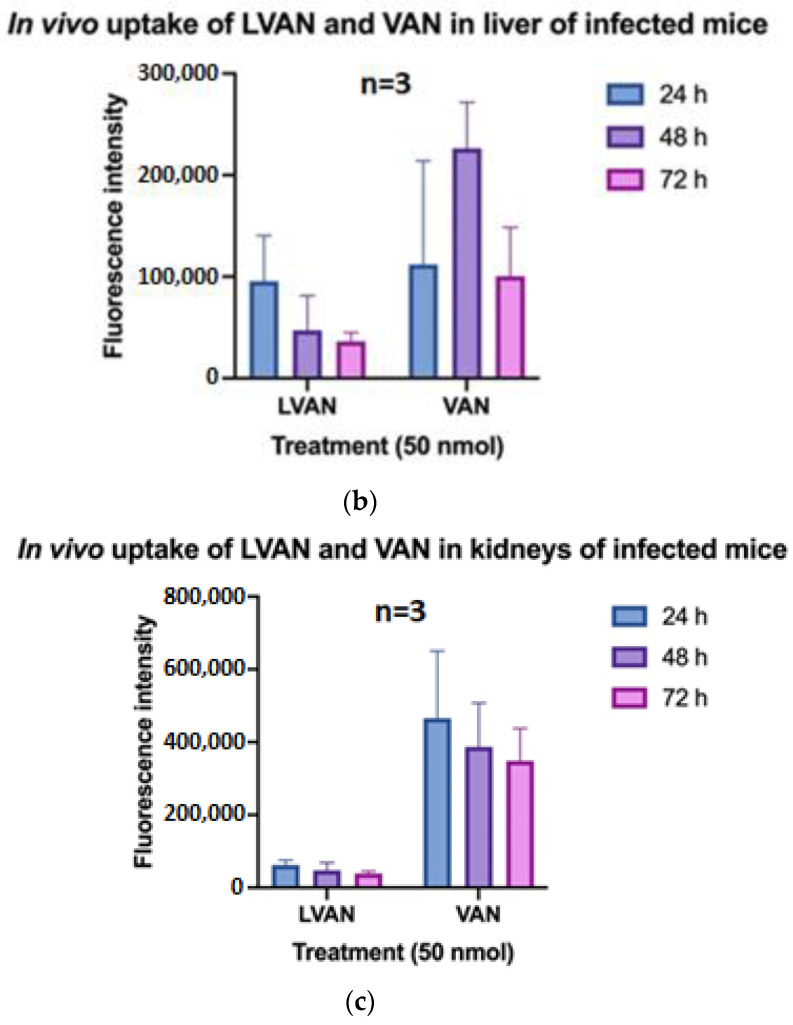
(**a**) The in vivo uptake of LVAn and VAN in MRSA infected mice thigh tissue is shown. Significant accumulation of LVAN over time, post-injection, as compared to VAN in MRSA-infected thigh tissue in mice model is shown. A significant improvement in the localization and retention of LVAN compared to VAN is seen at the infected thigh tissues. (**b**) The in vivo uptake of LVAn and VAN in liver of MRSA infected mice is shown. Compared to LVAN, VAN was retained in the liver for a longer time, with a significant accumulation of VAN at 48 h post-injection. The data reveal a promising safety profile of LVAN for further development. (**c**) The in vivo uptake of LVAn and VAN in kidneys of MRSA infected mice is shown. An insignificant accumulation of LVAN in the kidneys compared to VAN was seen at all time points studied, strengthening the idea of non-toxic delivery of the antibiotic via the liposomal delivery system.

Fluorescence intensity is directly proportional to the tissue uptake of VAN. LVAN accumulated at the infected tissue site for a prolonged time period of 72 h compared to VAN. There seemed to be no significant difference in the accumulation of the drug between LVAN and VAN treatment groups at 24 and 48 h. However, there was a marked difference in the intensity of rhodamine-VAN accumulation for LVAN group at 72 h, compared to VAN alone (Figure 6a).

The liver and kidney uptake of LVAN was observed to be relatively lower than VAN at every time point (Figure 6b,c). Thus, LVAN was retained at the site of infection for a prolonged duration of time. Liposomes are long-circulating nanoparticles that offer the benefit of sustained-release over conventional formulations [34]. LVAN improved the circulation half-life of VAN and eliminated the possibility of drug deposition in the liver and the kidneys. The data corroborates with previous results and solidifies our hypothesis that liposomal vancomycin can deliver the drug at the target infection site for a prolonged time, possibly either eliminating or drastically reducing VAN-associated nephrotoxicity.

#### 3.6.2. In Vivo Antibacterial Efficacy Study

**Figure 7:**
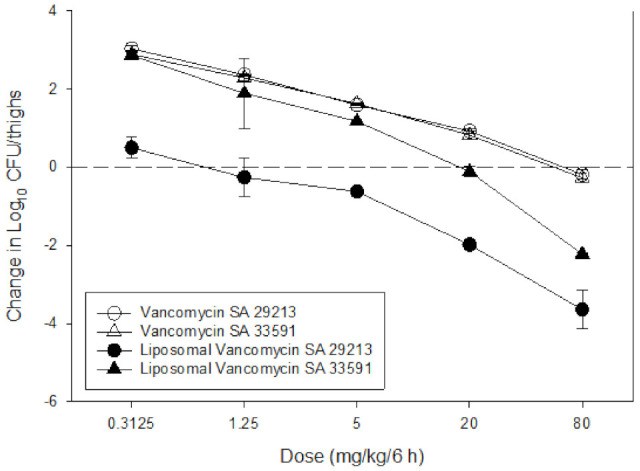
Superior bactericidal effect of LVAN in mice thigh infection model. LVAN shows bactericidal effect at the dose of 1.25 and 20 mg/kg/6 h for MSSA and MRSA, respectively. VAN shows bactericidal effect at a much higher dose of 80 mg/kg/6 h for both MSSA and MRSA. This data strongly support the rationale for developing targeted LVAN-As for treating MRSA.

VAN is the first-line treatment of MRSA, however, poor plasma half-life and tissue distribution have limited its overall efficacy in the treatment of infection [24,35]. Our multi-pronged FR-targeting LVAN showed the enhanced plasma circulation and the selective accumulation of nano-antibiotics at the site of infection. The dose–response curves for LVAN versus VAN suggest that LVAN showed better killing activity compared to free drug VAN in both MSSA and MRSA. Notably, LVAN was able to show bacteriostatic activity at a dose of 0.3125 mg/kg/6 h and bactericidal activity at a dose of 1.25 mg/kg/6 h and higher doses in MSSA strain 29213. For MRSA strain 33591, the bacteriostatic dose was observed to be 20 mg/kg/6 h, and bactericidal activity was observed at a dose of 40 mg/kg/6 h (Figure 7).

## 4. Conclusions

The current study involves the development and characterization of folate targeted liposomal formulation to encapsulate vancomycin. The formulation was tested in vitro and in vivo for antibacterial efficacy against MRSA and tissue uptake in various organs in comparison to the free vancomycin. The liposomes were able to show higher bactericidal effects compared to free VAN, controlled release of vancomycin, and reduced kidney accumulation within the therapeutic range of vancomycin. The folate-receptor targeted liposome of vancomycin LVAN is a promising candidate to overcome the limitation of conventional treatment of vancomycin by enhancing the delivery of vancomycin to the MRSA infected tissue with better bactericidal activity and reducing the accumulation of vancomycin in the kidney, portending promising potentials for further development.

## Figures and Tables

**Table 1:** MIC values of VAN and LVAN for strains 494 and 29213.

Strain	MIC Values (mg/L)	
	VAN	LVAN
494	1	0.5–1
29213	1	0.5–1
N315	0.5	0.5

**Table 2:** Zone of inhibition (in mm) for different strains of MRSA and MSSA.

Strain	Zone of Inhibition (mm)	
	VAN	LVAN
494	10.86	11.66
29213	10.37	11
N315	12.86	12.6
